# Multidomain Cognitive Decline and Motor Disability in Multiple Sclerosis: A Retrospective Cohort Study

**DOI:** 10.3390/jcm15197627

**Published:** 2026-10-01

**Authors:** Giuseppe Romano, Maria Cropano, Mario Fordellone, Simona Raimo, Cinzia Coppola, Deborah Archetto, Lidia Ammendola, Daniela Malangone, Simona Bonavita, Giacomo Lus, Elisabetta Signoriello

**Affiliations:** 1 Multiple Sclerosis Center, UOSD Second Neurology, University of Campania “Luigi Vanvitelli”, 80131 Naples, Italy; giuseppe.r1495@gmail.com (G.R.); simona.bonavita@unicampania.it (S.B.); giacomo.lus@unicampania.it (G.L.); 2 Department of Health Sciences, “Magna Graecia” University of Catanzaro, 88100 Catanzaro, Italy; 3 Medical Statistics Unit, University of Campania “Luigi Vanvitelli”, 80138 Naples, Italy; 4 Department of Medical and Surgical Sciences, “Magna Graecia” University of Catanzaro, 88100 Catanzaro, Italy; simona.raimo@unicz.it

**Keywords:** multiple sclerosis (MS), cognitive impairment (CI), progression independent of relapse activity (PIRA)

## Abstract

**Background:** Multiple sclerosis (MS) is characterized by heterogeneous trajectories of motor and cognitive disability. This study aimed to characterize longitudinal cognitive and motor trajectories across MS phenotypes and to identify baseline predictors of subsequent worsening. **Methods:** This retrospective cohort included 68 adults with MS followed for approximately six years. Cognitive performance was assessed at baseline (T0) and follow-up (T1) using the Rao’s Brief Repeatable Battery (BRB-N) and quantified using the Cognitive Impairment Index (CII). Clinical, demographic, neuropsychological, psychological, and neuroimaging variables were collected. Longitudinal cognitive changes were evaluated using linear mixed-effects models including baseline phenotype, time, and phenotype-by-time interaction. Logistic regression was used to investigate predictors of motor and cognitive worsening. **Results:** At baseline, 52.9% of patients met criteria for cognitive impairment. During follow-up, 50.0% experienced motor worsening and 77.9% experienced cognitive worsening; 44.1% met the study definition of cognitive progression independent of relapse activity. Memory CII increased significantly over time in the whole cohort (*p* = 0.003), whereas the phenotype-by-time interaction was not significant for Memory CII (*p* = 0.546), Executive CII (*p* = 0.211), or Total CII (*p* = 0.293). After Holm correction, the increase in Memory CII remained significant in baseline RRMS (+1.59; adjusted *p* = 0.007), but not in baseline SPMS (+1.40; adjusted *p* = 0.072) or PPMS (+0.57; adjusted *p* = 0.467). Eleven patients (32.4% of baseline RRMS) transitioned to SPMS and were retained in the baseline RRMS group for the primary longitudinal analyses. Their mean ΔEDSS was +1.91. **Conclusions:** Cognitive and motor progression represent partially distinct dimensions of MS evolution. Memory was the cognitive domain showing the clearest longitudinal worsening, particularly among patients classified as RRMS at baseline, while the magnitude of cognitive change did not differ significantly between baseline phenotypes.

## 1. Introduction

Multiple sclerosis (MS) is a chronic, neurodegenerative, immune-mediated disorder of the central nervous system that leads to physical, cognitive, and emotional impairment [1]. According to the 2013 revised Lublin classification, three clinical courses are recognized: relapsing–remitting MS (RRMS), which may progress to secondary progressive MS (SPMS), and primary progressive MS (PPMS), characterized by steady progression from onset [2]. Current evidence indicates that all MS phenotypes exhibit both inflammatory and neurodegenerative processes from the earliest stages, though to varying degrees, contributing to the diverse disease trajectories observed among patients [3]. While MS often presents with motor or sensory symptoms, cognitive impairment (CI) is highly prevalent, affecting 34–65% of adults. CI may occur abruptly during relapses or develop gradually over time, and it is present even in very early phases such as clinically isolated syndrome (CIS) and radiologically isolated syndrome (RIS). Its frequency increases in progressive forms. The most affected domains are processing speed, working memory, visuospatial abilities, and executive functioning [4,5]. Neuropsychiatric conditions, including depression and apathy, are common and significantly influence quality of life. Both are associated with cognitive dysfunction, and apathy may act as an early predictor of CI [6,7]. Cognitive decline results from combined white and grey matter damage. Grey matter atrophy—especially in the thalamus, hippocampus, and cortex—is strongly linked to cognitive deterioration. Functional MRI studies show disrupted connectivity, initially compensatory but eventually maladaptive [8]. Initial cognitive screening can be conducted with BICAMS, and if abnormalities are detected, a more comprehensive assessment is typically performed using the BRB-N battery [9,10]. Longitudinal studies show mixed findings: some report decline over short (2-year) or long (7-year) periods, whereas others suggest stability for several years, with deterioration becoming more evident after 10–20 years [11,12,13]. The study aimed to (1) characterize longitudinal cognitive and motor trajectories across baseline RRMS, SPMS, and PPMS; (2) assess whether the magnitude of cognitive change differed by baseline phenotype; and (3) identify baseline clinical, demographic, and cognitive factors associated with subsequent motor and cognitive worsening.

## 2. Methods

### 2.1. Study Design and Participants

This retrospective cohort study included patients with MS recruited between 2014 and 2023 at the Multiple Sclerosis II Neurology Centre of A.O.U. Luigi Vanvitelli, Naples. Eligible participants met the 2010 McDonald diagnostic criteria, were aged 18–65 years and had undergone at least two standardized neuropsychological assessments. Patients with MS activity, defined as either a clinical relapse or MRI activity consisting of new T2 lesions or gadolinium-enhancing lesions occurring within six months before cognitive testing, or severe medical or psychiatric conditions that could compromise the reliability of neuropsychological assessment were excluded. Clinical phenotype at baseline (T0) and conversion from RRMS to SPMS at follow-up (T1) was defined according to the 2013 revised Lublin criteria and based on sustained disability progression independent of relapse activity. Patients who converted from RRMS to SPMS were retained in the baseline RRMS group for the primary analyses and were additionally described as a separate subgroup in exploratory analyses.

### 2.2. Clinical, Demographic, Neuropsychological Assessment and Ecological Variables

Clinical and demographic information included sex, age, disease duration, functional system involved at disease onset, education, employment, marital status, parenthood, cognitive reserve, and disease-modifying therapy. EDSS was recorded at T0 and T1. Baseline MRI variables included the number of brain and spinal cord lesions; a brain lesion burden of more than nine lesions was categorized as high. During follow-up, annualized relapse rate (ARR) and annual new T2 lesion counts were recorded. Cognitive performance was assessed at T0 and T1 using the Rao’s Brief Repeatable Battery (BRB-N), a standardized neuropsychological battery for MS. The battery included tests of verbal learning and memory (Selective Reminding Test—Long-Term Storage and Consistent Long-Term Retrieval, with Delayed Recall), visuospatial learning and memory (10/36 Spatial Recall Test, including delayed recall), processing speed and attention (Symbol Digit Modalities Test), working memory and divided attention (Paced Auditory Serial Addition Test, 2- and 3-s versions), and verbal fluency (Word List Generation). Cognitive performance was converted to z-scores using normative data, and each test was graded according to deviation from the control mean [14]. Baseline cognitive impairment (CI) was defined as at least three tests scoring at grade 3. Composite Cognitive Impairment Indices were calculated for memory, executive functions, and total cognition. Cognitive change was assessed from the difference between T0 and T1 scores. Cognitive reserve was assessed using the Cognitive Reserve Index (CRI). Depressive symptoms were evaluated with the Beck Depression Inventory-II (BDI-II), and anxiety with the State-Trait Anxiety Inventory (STAI-Y1 and STAI-Y2). Cognitive progression independent of relapse activity (cognitive PIRA) was defined as longitudinal cognitive worsening occurring in the absence of clinical relapses or new T2 MRI lesions during follow-up.

### 2.3. Ethics

The study protocol was discussed and approved by the Scientific Committee and Ethics Committee of the University of Campania Luigi Vanvitelli (Prot. 12478/20). Written informed consent was obtained from all participants. The current report contains no individual or identifying information. Anonymized data may be shared upon reasonable request from a qualified investigator for replication of the reported procedures and results, provided that data transfer complies with European Union data-protection legislation.

### 2.4. Statistical Analysis

Continuous variables were summarized as mean and standard deviation or median and interquartile range (IQR), according to distribution assessed using the Shapiro–Wilk test. Categorical variables were summarized as absolute frequencies and percentages. For longitudinal analyses, linear mixed-effects models (LMMs) were used with CII domain as the dependent variable, baseline phenotype (RRMS, SPMS, PPMS), time (T0, T1), and phenotype-by-time interaction as fixed effects, and a subject-specific random intercept to account for repeated measurements. Importantly, phenotype was fixed according to baseline classification and was not redefined at follow-up. The phenotype-by-time interaction was used to test whether longitudinal cognitive change differed between baseline phenotypes. Between-phenotype comparisons at individual time points were performed using analysis of variance with Welch’s correction when appropriate for unequal group variances and sample sizes, followed by pairwise comparisons. Within-phenotype comparisons across time were evaluated using paired analyses. *p*-values for multiple pairwise comparisons were adjusted using the Holm method. Logistic regression was used to investigate predictors of motor worsening and cognitive worsening. Univariate analyses were followed by multivariable models including variables showing statistical significance in univariate testing. Odds ratios (ORs) and 95% confidence intervals (CIs) were reported where available. A two-sided *p*-value ≤ 0.05 was considered statistically significant. No formal a priori power analysis was performed because of the retrospective nature of the cohort and the availability of eligible patients with paired neuropsychological assessments. Phenotype-specific analyses, particularly those involving PPMS and the RRMS-to-SPMS converter subgroup, may therefore be underpowered to detect modest effects. Statistical analyses were conducted using R statistical software (version 4.1.3; 10 March 2022).

## 3. Results

### 3.1. Baseline Patient Characteristics

**Figure 1:**
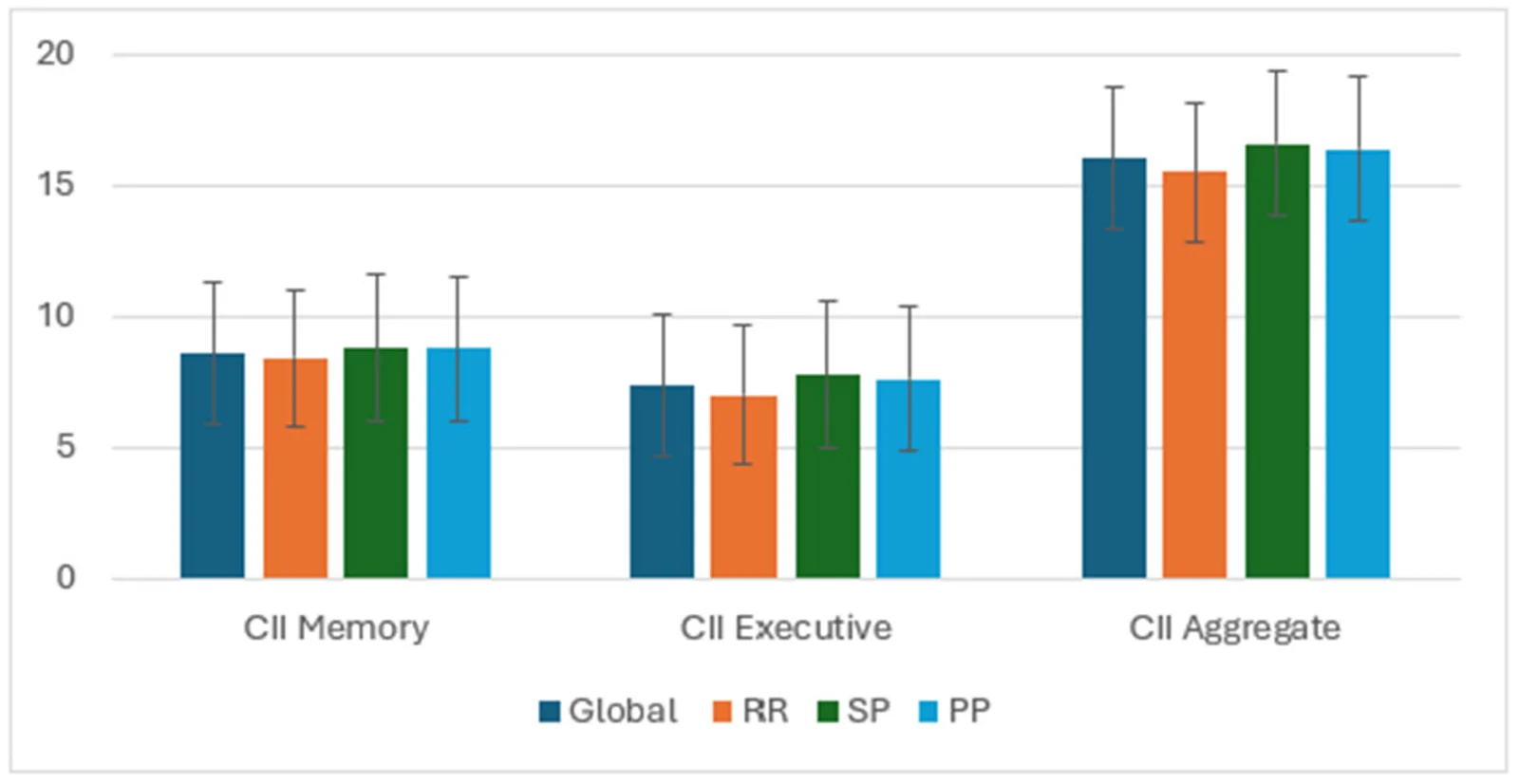
CII across different phenotype at baseline.

A total of 68 patients with MS were included. Overall, 66.2% were women and the mean age at diagnosis was 38.1 ± 11.8 years. Spinal onset was observed in 61.8% of patients. At T0, the phenotype distribution was 34 RRMS (50.0%), 20 SPMS (29.4%), and 14 PPMS (20.6%). Mean age at T0 was 46.6 ± 11.0 years and mean disease duration was 8.5 ± 8.11 years. At baseline, 52.9% of patients met the study definition of cognitive impairment. Mean CII values were 8.62 ± 3.62 for memory, 7.38 ± 2.47 for executive functions, and 16.0 ± 5.46 for total cognition. Baseline psychological measures included BDI-II 18.5 ± 12.4, STAI-Y1 45.9 ± 12.0, STAI-Y2 47.6 ± 11.4, and CRI 48.9 ± 11.6. Employment was reported in 32.4% of patients, 76.5% were married, and 64.7% had children. Additionally, Table 1 shows and compares baseline characteristics across three different disease phenotypes, highlighting statistically significant differences, while the histogram in Figure 1 displays the CII across the various phenotypes.

### 3.2. Follow-Up Patient Characteristics

The mean follow-up interval was 6.25 ± 1.39 years. At T1, mean age was 52.8 ± 11.4 years and mean EDSS was 4.41 ± 2.33. Mean ARR during follow-up was 0.162 ± 0.371 and the mean number of new T2 lesions was 1.24 ± 2.07. At T1, 57.4% of patients met criteria for cognitive impairment. Mean CII values were 9.94 ± 2.98 for memory, 7.18 ± 3.36 for executive functions, and 17.1 ± 5.55 for total cognition. Follow-up psychological measures revealed the following mean scores: BDI 13.6 ± 9.5, STAI-Y1 41.5 ± 9.47 and STAI-Y2 43.8 ± 10.5. Table 2 summarizes the main demographic, clinical, and cognitive characteristics of the study population at baseline and follow-up.

### 3.3. Motor Progression

**Figure 2:**
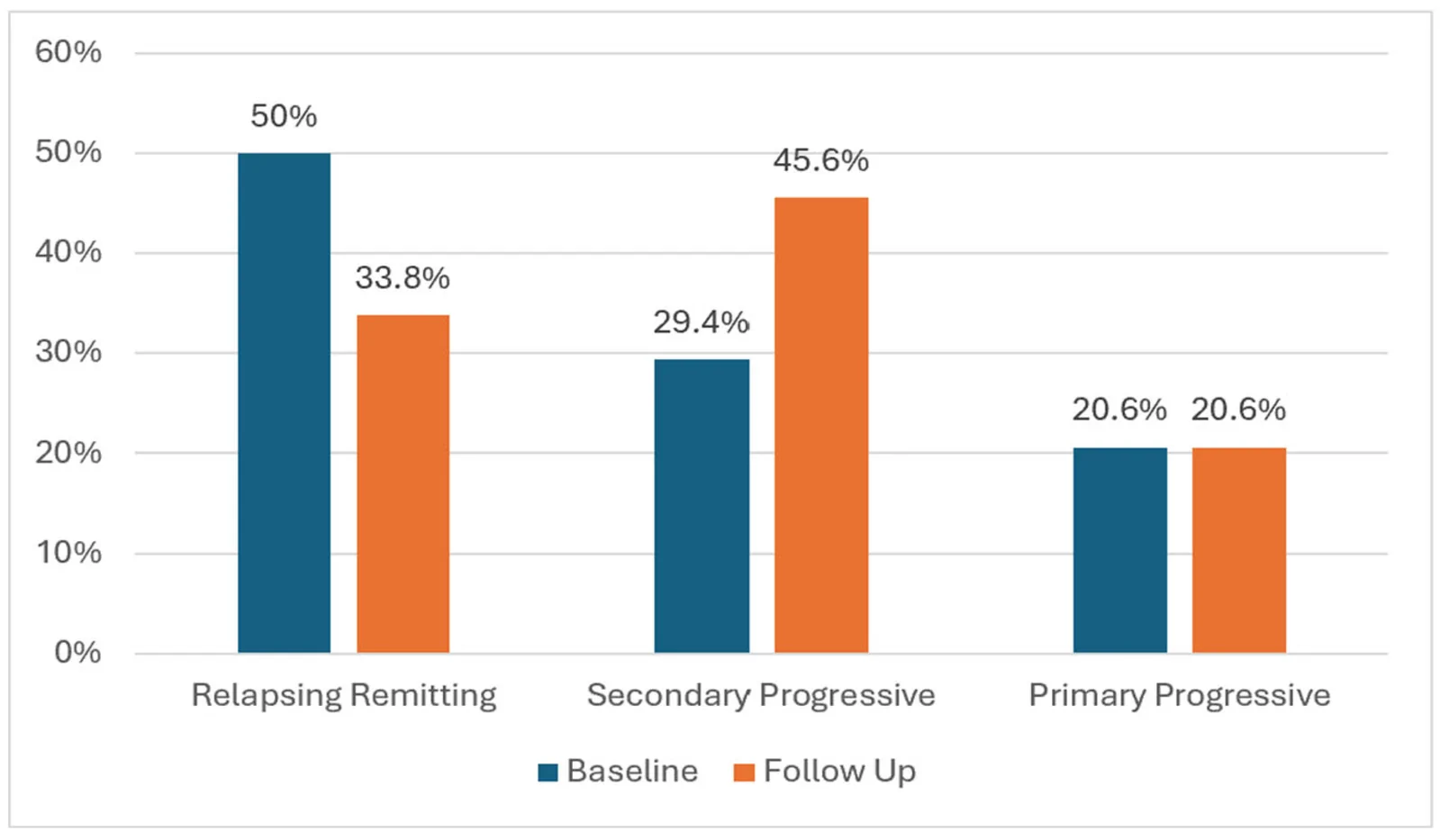
Comparative bar chart showing the percentage of patients with RRMS, PPMS and SPMS at baseline and follow up.

During follow-up, 50.0% of patients experienced motor deterioration, with a mean EDSS increase of 0.55 ± 1.00. To better illustrate the longitudinal changes in disease course Figure 2 presents a comparative bar chart showing the percentage of individuals classified as RR, PP, and SP at both baseline and follow-up evaluations. Motor deterioration is more prevalent among SPMS patients (75.0%), followed by PPMS (57.1%) and, to a lesser extent, RRMS (32.4%), with a statistically significant difference (*p* = 0.01). Eleven patients (32.4% of the baseline RRMS group) subsequently transitioned to SPMS according to the prespecified definition. This subgroup showed marked motor deterioration, with a mean ΔEDSS of +1.91 ± 1.16. RRMS patients who did not transition showed a mean ΔEDSS of −0.23 ± 0.68. Follow-up duration and ARR did not differ significantly between the descriptive groups.

### 3.4. Cognitive Progression and Longitudinal Changes in CII Across MS Phenotypes

**Figure 3:**
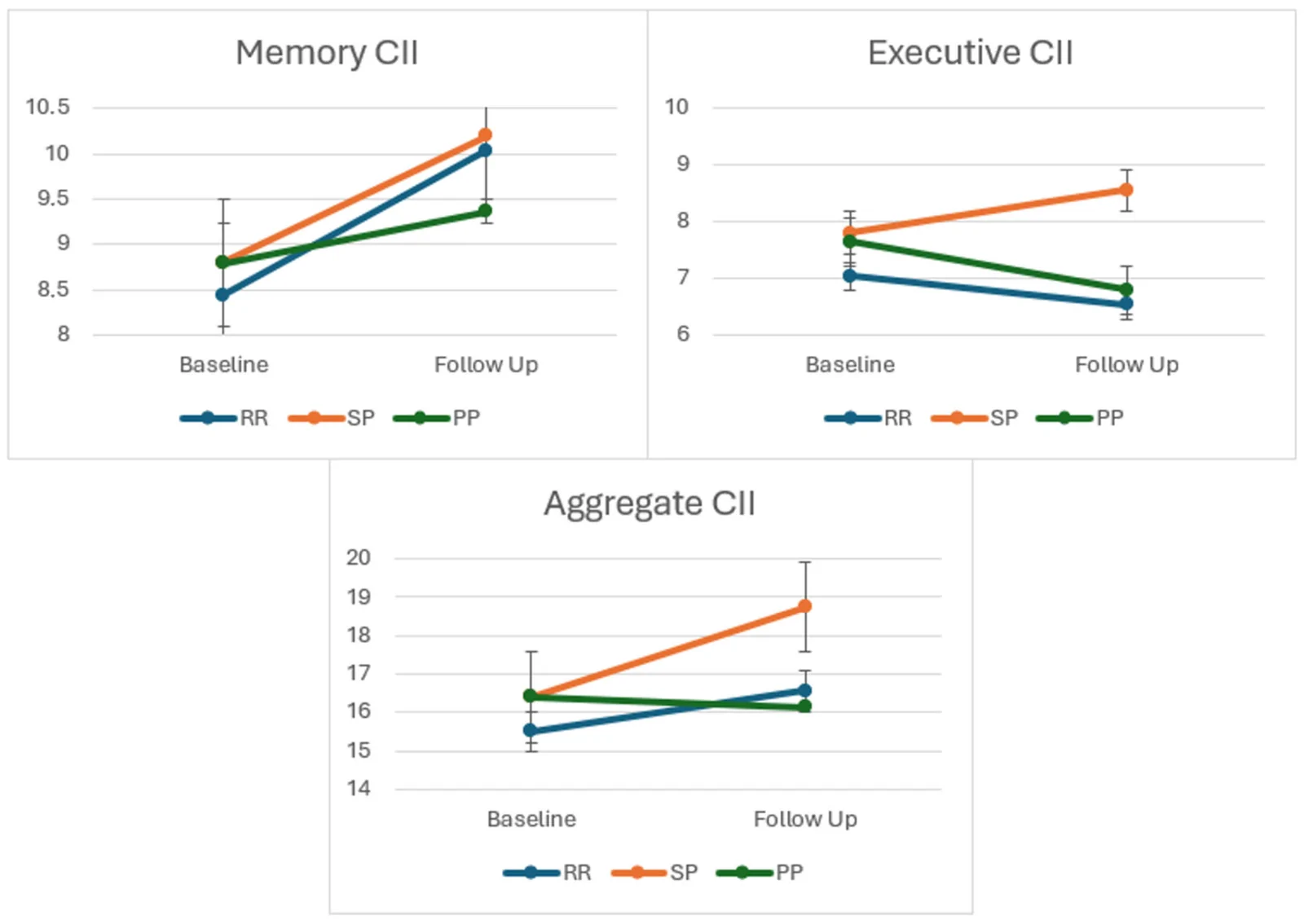
Memory, executive and aggregate CI trends across phenotype at baseline and follow-up.

Overall, 77.9% of patients experienced cognitive worsening during follow-up, and 44.1% met the study definition of cognitive PIRA. In the LMMs, Memory CII showed a significant longitudinal increase over time (*p* = 0.003), indicating worsening memory impairment. The phenotype-by-time interaction was not significant for Memory CII (*p* = 0.546), Executive CII (*p* = 0.211), or Total CII (*p* = 0.293), indicating that the magnitude of longitudinal cognitive change did not significantly differ according to baseline phenotype. Figure 3 graphically represents longitudinal changes in CII for memory, executive functions, and global cognition. Post-hoc within-phenotype comparisons, with Holm correction, showed that the increase in Memory CII remained significant among baseline RRMS patients (+1.59; adjusted *p* = 0.007), whereas the corresponding changes did not reach the corrected significance threshold in baseline SPMS (+1.40; adjusted *p* = 0.072) or PPMS (+0.57; adjusted *p* = 0.467). These findings support a significant overall longitudinal memory decline without evidence of a statistically significant phenotype-specific difference in the trajectory.

### 3.5. Analysis of Cognitive and Motor Domains: An Examination of Associated Factors

To gain deeper insight into the factors influencing cognitive and motor outcomes within our cohort, we first conducted univariate analyses to explore the association of a series of clinical and demographic variables—including age, sex, type of onset, disease phenotype, EDSS score at baseline, brain lesion load, aggregate CII at baseline, ARR, disease duration and ecological variables (marital status, employment, and parenthood)—with the two dichotomous outcomes of interest, namely motor worsening and cognitive worsening. Table 3 presents the results in the univariate analyses for motor and cognitive worsening. Statistical significant variables were subsequently entered into multivariate logistic regression models to identify independent predictors of these outcomes. The multivariate analysis for motor worsening identified parenthood as a protective factor (*p* = 0.004, OR = 0.104, IC 0.02–0.49), while baseline EDSS (*p* = 0.047, OR = 2.011, IC 1–4) emerged as negative predictor. While, the multivariate analysis for cognitive worsening revealed a statistically significant association with higher aggregate CII values (*p* = 0.006, OR = 1.299, IC 1.07–1.56) as negative predictive factors, and female sex (*p* = 0.015, OR = 0.131, IC 0.025–0.67) employment status (*p* = 0.036, OR = 0.139, IC 0.01–0.48) as protective factor. Table 4 reports the results of the univariate analyses restricted to the RR patient group, with conversion to SP as the outcome, aimed at identifying potential predictive factors. In the multivariate model, high value of aggregate CII showed a trend towards significance (*p* = 0.061, OR = 1.2305, IC 0.99–1.52).

## 4. Discussion

This retrospective study makes a significant contribution to the understanding of cognitive and motor trajectories of the disease, with a mean follow-up over six years. The findings confirm the complex and multidimensional nature of MS, particularly with regard to the course of physical disability and cognitive decline, which is characterised by an interplay between inflammatory, neurodegenerative, and neuronal compensatory processes [15]. In the context of MS, the progression of motor disability represents one of the most clinically relevant aspects, with considerable impact on both functional status and prognosis. Although the underlying mechanisms of progression are heterogeneous and multifactorial, it is evident that disability accumulation does not follow a linear trajectory but tends to accelerate around the transition from relapsing–remitting forms to progressive forms [16]. In our cohort, clinically meaningful motor worsening was observed in approximately 50% of patients over a mean follow-up of six years, with an average EDSS increase of 0.55 ± 1. This finding is consistent with the literature, suggesting that motor disability is an active process already in the early stages, but becomes more pronounced during the progressive phases of the disease [17]. An analysis of clinical phenotypes reveals a clear gradient in disability progression: patients with RR forms show a relatively stable motor profile over time; in contrast, individuals with PP and SP forms show significantly higher rates of deterioration (0.75 ± 0.61 and 0.57 ± 0.43) [18]. However, it is the subgroup of RR patients who transition to the SP form that shows the most severe trajectory, with a mean EDSS increase of 1.91 ± 1.16 and a phenotypic conversion observed in 32.3% of cases. This pattern supports the hypothesis that the transition phase represents a critical point in the course of MS, marking the shift from episodic and partially reversible disability to a persistent and irreversible accumulation [19]. The multivariate analysis identified baseline EDSS as a negative prognostic factor for motor worsening, confirming its established role as a predictor of disability progression. Conversely, parenthood emerged as a protective factor. While the underlying mechanisms remain uncertain, this association may reflect psychosocial influences such as healthier lifestyles, or parenthood as a surrogate marker of preserved functional reserve, but the relationship may also be bidirectional: better clinical stability and well-being may increase the likelihood of having children. Although scarcely addressed in the literature, ecological and social determinants of prognosis merit further exploration. Unexpectedly, factors such as functional system involvement at onset and spinal lesion count, significant in univariate models, did not retain significance in multivariate analysis. This discrepancy, contrasting with previous reports as well as the higher proportion of spinal onset disease, may reflect sample heterogeneity or limited statistical power [20]. Focusing on the subgroup of RR patients who converted to SP MS, baseline factors such as higher aggregate CII tended to be associated with an increased likelihood of progression. Once again, baseline EDSS and spinal lesion count, although significant in univariate analyses, did not emerge as independent predictors in the multivariate model. This may reflect the relatively small sample size [20]. Previous studies have indicated that brain lesion burden, cortical thinning, and thalamic atrophy are major contributors to disability at intermediate EDSS levels, whereas spinal cord pathology predominates in driving motor impairment at more advanced stages [21]. In our cohort, motor worsening was not associated with imaging parameters, likely due to methodological and population-related factors. Conventional lesion counting, rather than volumetric or advanced MRI, may have limited the sensitivity to subtle changes, especially given the clinical heterogeneity of our cohort. Cognitive Involvement in MS emerges as a clinically relevant aspect from the early stages of the disease, often progressing insidiously yet steadily. Overall, these findings suggest that although cognitive impairment is already present in the early stages, deterioration becomes more pronounced and clinically apparent during the transition to the SP phase [22]. In our cohort, already at baseline more than half of the patients (52.9%) exhibited cognitive impairment—a figure that rose to 57.4% at follow-up, marking an overall increase of 9.5%. This finding, consistent with the literature, confirms that cognitive decline is a widespread phenomenon even in RR forms and not exclusive to progressive phases [6]. The longitudinal assessment of key cognitive domains revealed a significant deterioration in memory across the entire cohort between baseline and follow-up, particularly in RRMS patients, whereas executive and global domains remained relatively stable over time. However, the magnitude of change did not statistically differ among baseline RRMS, SPMS, and PPMS. These findings should therefore not be interpreted as evidence that RRMS has a uniquely progressive cognitive trajectory; rather, they indicate that memory was the most sensitive longitudinal cognitive domain in this cohort and that the available sample did not demonstrate a statistically different trajectory across baseline phenotypes. Previous studies have shown that the prevalence of cognitive impairment is largely comparable between SPMS and PPMS, with broadly similar neuropsychological profiles. To our knowledge, however, longitudinal data specifically addressing cognitive changes in PPMS remain limited. In our cohort, we observed, quite unexpectedly, that PPMS patients did not exhibit a significant decline in memory or executive functions over the follow-up period [23]. Multivariate analysis confirmed the predictive value of the CII, with higher baseline scores strongly associated with subsequent cognitive worsening, consistent with the notion that early deficits reflect reduced cognitive reserve. Identifying individuals with elevated CII values may therefore assist clinicians in risk stratification and prioritising cognitive monitoring and rehabilitation strategies. Female sex emerged as a significant predictor of lower risk of cognitive worsening, aligning with evidence that men often experience a more aggressive disease course, with faster progression and more severe cognitive deficits. Employment status was also associated with reduced risk of cognitive decline, likely reflecting the concept of cognitive reserve: professional activity stimulates higher-order cognitive functions, promotes social interaction, and maintains compensatory networks that buffer the impact of disease-related brain damage. Clinically, these findings underscore the importance of maintaining occupational and social engagement as part of strategies to preserve cognitive health in MS. Surprisingly, parameters such as lesion load did not show significant associations, even in univariate analyses. However, the lack of correlation between lesion burden and cognitive progression in our study may reflect methodological limitations, as lesion load was quantified through simple lesion counting, a technique with limited sensitivity compared to volumetric assessments. Memory and processing speed emerge as the most vulnerable cognitive domains. In particular, memory involvement is evident from the earliest stages in patients with a diagnosis of RRMS, as demonstrated by the early and marked involvement of the hippocampus [24]. Several limitations should be acknowledged. The retrospective, single-centre design, relatively small sample size, and unequal phenotype distribution may have limited statistical power. The use of the 2010 McDonald criteria, although appropriate for the period in which most patients were diagnosed, may also limit comparability with contemporary cohorts. Neuroimaging analyses were primarily based on lesion counts rather than volumetric or advanced MRI measures, potentially reducing sensitivity to subtle associations between structural damage and clinical or cognitive outcomes. Future prospective multicentre studies should include larger, phenotype-stratified cohorts, harmonized longitudinal cognitive assessments, advanced MRI measures, and biomarkers of neuroaxonal injury. More frequent assessments could help distinguish true cognitive decline from practice effects and clarify the temporal relationship between cognitive and motor progression, inflammatory activity, and RRMS-to-SPMS transition.

## 5. Conclusions

This longitudinal study demonstrates that motor and cognitive progression are related but distinct dimensions of MS evolution. Using baseline phenotype as a fixed grouping variable, memory CII showed significant worsening over time, but the phenotype-by-time interaction was not significant, indicating no demonstrable difference in the magnitude of cognitive change across baseline RRMS, SPMS, and PPMS. Patients who transitioned from RRMS to SPMS showed particularly marked motor deterioration. These findings support longitudinal monitoring that integrates sensitive cognitive and motor measures while avoiding phenotype reclassification as a source of bias. Larger prospective studies incorporating advanced MRI and biological markers are needed to identify patients at highest risk of multidimensional progression and guide personalised management strategies.

## Figures and Tables

**Table 1:** Baseline characteristics across three different disease phenotypes.

Characteristic	RRMS (n = 34)	SPMS (n = 20)	PPMS (n = 14)	*p*-Value
Female, n (%)	23 (67.6)	14 (70.0)	8 (57.1)	0.75
Age, median (IQR), y	45.20 (35.58–50.15)	51.45 (40.33–57.62)	53.50 (44.67–58.80)	0.03
Onset Functional System:				0.56
- Brainstem onset, n (%)	9 (26.5)	6 (30.0)	3 (21.4)	
- Spinal onset, n (%)	20 (58.8)	11 (55.0)	11 (78.6)	
- Visual onset, n (%)	5 (14.7)	3 (15.0)	0 (0)	
>9 brain T2 lesions, n (%)	14 (41.2)	3 (15.0)	3 (21.4)	0.11
EDSS, median (IQR)	2.0 (1.1–3.0)	6.0 (5.5–6.5)	5.0 (4.0–6.0)	<0.001

**Table 2:** Demographic and clinical characteristics at baseline and follow-up.

Characteristic	Baseline (n = 68)	Follow-Up (n = 68)
Age, mean ± SD, y	46.6 ± 11.0	52.8 ± 11.4
EDSS, mean ± SD	3.86 ± 1.98	4.41 ± 2.33
Cognitive impairment, n (%)	36 (52.9)	39 (57.4)
Memory CII, mean ± SD	8.62 ± 3.62	9.94 ± 2.98
Executive CII, mean ± SD	7.38 ± 2.47	7.18 ± 3.36
Total CII, mean ± SD	16.0 ± 5.46	17.1 ± 5.55

**Table 3:** Univariate analysis to verify clinical predictors of motor progression and cognitive deterioration.

Variable	Motor Univ. *p* Value	Motor Univ. OR, [95% IC]	Cognitive Univ. *p* Value	Cognitive Univ. OR, [95% IC]
Age	0.537	0.986 [0.94–1.03]	0.620	0.987 [0.93–1.04]
Female sex	0.798	0.877 [0.32–2.40]	0.004	0.163 [0.04–0.56]
System at onset, Brainstem				1.520
- Visual	0.272	0.382 [0.06–2.13]	0.621	1.560 [0.267–9.11]
- Spinal	0.34	0.579 [0.18–1.78]	0.329	[0.14–1.93]
Baseline phenotype, RRMS				
- SPMS	0.004	6.273 [1.18–21.70]	0.573	0.655 [0.14–2.85]
- PPMS	0.116	2.788 [0.77–10.02]	0.113	0.267 [0.05–1.37]
Baseline EDSS	0.001	1.617 [1.21–2.15]	0.053	0.734 [0.53–1.00]
Spinal lesion number	0.019	1.524 [1.07–2.16]	0.077	0.682 [0.44–1.04]
Brain lesion number	0.011	4.579 [1.42–14.69]	0.138	3.343 [0.67–16.44]
ARR during follow-up	0.329	0.514 [0.13–1.95]	0.650	1.406 [0.32–6.12]
New T2 lesions during FU	0.298	0.879 [0.69–1.12]	0.529	1.086 [0.84–1.40]
Disease duration	0.505	0.98 [0.92–1.04]	0.243	0.949 [0.87–1.03]
Marital status	0.257	0.514 [0.16–1.62]	0.315	1.909 [0.54–6.74]
Employment	0.605	0.764 [0.27–2.12]	0.003	0.146 [0.04–0.51]
Parenthood	0.046	0.346 [0.12–0.97]	0.300	1.83 [0.57–5.95]
Aggregate CII	0.036	0.904 [0.82–0.99]	0.043	1.138 [1.003–1.291]

**Table 4:** Univariate analysis to identify clinical predictors of conversion from RR to SP MS.

Variable	Motor Progression Univ. *p* Value	Motor Univ. OR, [95% IC]
Age	0.078	0.92 [0.83–1.01]
Female sex	0.73	10.766 [0.168–3.49]
System at onset, Brainstem		
- Visual	0.172	0.190 [0.01–2.06]
- Spinal	0.666	0.667 [0.10–4.20]
Baseline EDSS	0.02	2.427 [1.15–5.11]
Spinal lesion number	0.028	2.161 [1.08–4.29]
Brain lesion number	0.073	4.91 [0.86–27.88]
ARR during follow-up	0.811	1.25 [0.20–7.75]
New T2 lesions during FU	0.348	0.944 [0.83–1.06]
Disease duration	0.505	0.98 [0.92–1.04]
Marital status	0.370	2.06 [0.42–9.97]
Employment	0.714	1.309 [0.31–5.53]
Parenthood	0.278	0.444 [0.103–1.92]
Aggregate CII	0.009	1.130 [1.07–1.59]

## Data Availability

The data supporting the findings of this study are not publicly available due to privacy and confidentiality considerations but are available from the corresponding author upon reasonable request, subject to applicable regulations and institutional policies.
